# Effects of Resistance Circuit-Based Training on Body Composition, Strength and Cardiorespiratory Fitness: A Systematic Review and Meta-Analysis

**DOI:** 10.3390/biology10050377

**Published:** 2021-04-28

**Authors:** Domingo Jesús Ramos-Campo, Luis Andreu Caravaca, Alejandro Martínez-Rodríguez, Jacobo Ángel Rubio-Arias

**Affiliations:** 1 Department of Education, University of Alcalá, 28085 Madrid, Spain; 2 Sport Science Faculty, Catholic University of Murcia, 30107 Murcia, Spain; landreu@ucam.edu; 3 International Chair of Sport Medicine, Catholic University of Murcia, 30107 Murcia, Spain; 4 Department of Analytical Chemistry, Nutrition and Food Sciences, Faculty of Sciences, University of Alicante, 03690 Alicante, Spain; amartinezrodriguez@ua.es; 5 LFE Research Group, Department of Health and Human Performance, Faculty of Physical Activity and Sport Science-INEF, Technical University of Madrid, 28040 Madrid, Spain; jacobo.rubio2@gmail.com; 6 Department of Education, University of Almería, 04120 Almeria, Spain

**Keywords:** fat mass, maximum oxygen uptake, muscle mass, one maximum repetition

## Abstract

**Simple Summary:**

Resistance circuit-based training is an effective training method to decrease total body fat and increase muscle mass in adults. Resistance circuit-based training promotes concurrent improvements in strength performance and cardiorespiratory fitness variables in adults. If training load is managed properly, a greater effect on body composition and strength adaptations is possible. Regarding 1-RM improvements, the effect of this type of training is influenced by the training status, obtaining greater effects in untrained and active adults, and by the training characteristics, showing a larger effect in protocols with three sessions and a duration of >20 training sessions. For body fat mass decreases, the effect of resistance circuit-based training was significantly greater in protocols that used low (<60% 1-RM) or moderate intensity (60–80% 1-RM) and short periods or rest between exercises (10–30 s).

**Abstract:**

We assessed the effects of resistance circuit-based training (CT) on strength, cardiorespiratory fitness, and body composition. A systematic review with meta-analysis was conducted in three databases, ending on March, 2020. Meta-analysis and subgroup analysis were used to analyze the effects of pre–post-intervention CT and differences from control groups (CG). Of the 830 studies found, 45 were included in the meta-analysis (58 experimental groups (*n* = 897) and 34 CG (*n* = 474)). The CT interventions led to increases in muscle mass (1.9%; *p* < 0.001) and decreases in fat mass (4.3%; *p* < 0.001). With regard to cardiorespiratory fitness, CT had a favorable effect on VO2max (6.3%; *p* < 0.001), maximum aerobic speed or power (0.3%; *p* = 0.04), and aerobic performance (2.6%; *p* = 0.006) after training. Concerning strength outcome, the CT increased the strength of the upper and lower extremities. Only the magnitude of strength performance appears to be influenced by the training (number of sessions and frequency) and the training status. Moreover, low and moderate intensities and short rest time between exercise increase the magnitude of change in fat mass loss. Therefore, CT has been shown to be an effective method for improving body composition, cardiorespiratory fitness, and strength of the lower and upper limbs.

## 1. Introduction

Many guidelines for physical activity and exercise published by international associations [1] have recommended an increase in maximal strength, along with cardiovascular fitness and the improvement of body composition, to provide overall health benefits in the young [2] and elderly population [3], as well as for the improvement of quality of life in patients with different pathologies such as cancer [4], kidney diseases [5], or diabetes [6]. In numerous pathologies and in athletic disciplines, endurance and resistance training are often trained concomitantly (i.e., concurrent training) as part of a periodized training program [7]. To date, one of the most common concurrent training methods is resistance circuit-based training (CT) [8], which promotes aerobic conditioning, muscular endurance, and neuromuscular and strength adaptations in one workout [9]. This method comprises single or several sets of different exercises completed in succession with little rest between exercises. The exercises are performed at low (<60% of one maximum repetition (1-RM)), moderate (60–80% 1-RM), or high loads (>80% 1–RM), with a high number of repetitions (12–15) or lower (<12) or using a set length of time (e.g., 30 s), and with a very short rest period between exercises (e.g., 30 s) [8]. CT has been previously recommended as an elective type of training in untrained or people with a lower basal level of fitness [10], due to fact that this type of training can improve both upper body maximal strength and VO_2_max [8]. In addition, CT has been applied as an introductory type of training for developing cardiovascular conditioning and 1-RM in athlete periodization programs [8]. However, the results of CT in trained athletes are controversial, because some studies [10] conclude that the aerobic adaptations may be lower in those who are more fit, while other CT studies find using heavier loads [11,12] for developing strength and cardiorespiratory fitness are much more likely to improve them.

During CT training, athletes elicit higher heart rate but similar strength requirements to those reported during a traditional strength-training session [13]. In addition, in comparison to traditional strength training, higher lactate concentrations and ratings of perceived exertion have been shown during CT [14]. Moreover, CT has been shown to elicit higher oxygen consumption during the session in comparison to a traditional strength-training session [15] and aerobic treadmill exercise [9]. Furthermore, greater excess post-exercise oxygen consumption during the recovery has been reported after CT compared to a traditional resistance training session [16] or a treadmill exercise [17,18]. These characteristics of CT are typically related with the specific strength and aerobic adaptations and body composition changes linked to this type of training. In terms of cardiorespiratory fitness, previous studies have observed improvements in VO_2_max [19,20,21,22] and endurance performance [20,23,24]. In addition, CT increases strength performance [11,19,25] and optimizes body composition [11,25]. However, other researchers do not find significant improvements in maximal strength [10], muscle growth [26,27], aerobic performance [12], or VO_2_max [12,28], or significant decreases in body fat [10,26] after a CT training program in healthy adults.

These controversial findings regarding the effectiveness of CT may be related to the training characteristics applied during the different CT training protocols [8]. For instance, the load intensity (i.e., high vs. low loads), number of rounds, number of sets, number of repetitions, and number of exercises or rests between exercises must be manipulated in the CT training protocol; these characteristics establish a different dosage of training, and consequently, they impact on the interference effect and promote different endurance and strength adaptations. Therefore, little is known about the optimal CT dose for enhancing endurance and strength, and to optimize body composition, or about what the more effective CT characteristics are (program duration, number of sets, rounds, repetitions, rest, training intensity, etc.). Thus, to obtain an answer to these aforementioned questions and to determine the most effective CT dose in order to optimize the body composition and improve strength and cardiorespiratory fitness, it is necessary to analyze the quantitative, meta-analytical, and meta-regression effect of the current literature.

A previous systematic literature review with meta-analysis [8] has critically discussed the potential of CT to increase VO_2_max and upper body 1-RM. A potential limitation of this previous work is that it did not include lower body maximal strength. Therefore, to the best of our knowledge, no study has analyzed the overall effect of CT on whole-body strength training. Another limitation of this previous work is that it only analyzed VO_2_max as a cardiorespiratory fitness variable, and previous studies [29,30] confirmed that other variables, such as maximum aerobic speed (MAS), are considered important indicators of aerobic performance that may in turn be useful for prescribing individualized training zones. Remarkably, no previous study has systematically analyzed the effect of CT on body composition variables, despite this training method being commonly used as an intervention to lose body mass and fat mass and to increase muscle mass [31]. Taken as a whole, this clearly demonstrates the growing interest around CT’s potential and the need to conduct new analysis. Therefore, our aim was to perform a systematic review and meta-analysis to investigate the use of resistance circuit-based training to maximize body composition and improve strength and cardiorespiratory fitness markers in healthy adults. A secondary aim was to examine which training characteristics and dosage produced the greatest adaptations of the variables analyzed. 

## 2. Materials and Methods

### 2.1. Design

The methodological process was based on the recommendations formulated in the Preferred Reporting Items for Systematic Reviews and Meta-Analyses (PRISMA) declaration [32]. Additionally, the review was registered in PROSPERO International Prospective Register of Systematic Reviews (www.crd.york.ac.uk/prospero/index.asp (accessed on 1 February 2019), identifier CRD42019122373).

### 2.2. Data Sources and Search Profile

A comprehensive literature search was performed using PubMed-Medline, Web of Science, and the Cochrane Library, from database inception up to March, 2020. A database search for scientific articles related to resistance circuit-based training and its effects on endurance performance, strength, and body composition was performed. Two different authors (JARA and DJRC) performed the search independently, and the results of the search between the authors were the same. The following combination of terms was used: “circuit training” or “circuit weight training” or “circuit strength training” or “circuit resistance training”. The Boolean operator “and” was used to combine these descriptors with: “body composition” or “endurance” or “oxygen consumption” or “strength” or “resistance”.

### 2.3. Selection Criteria

No restrictions for the search date were applied. The specific inclusion criteria were: (1) studies examining CT intervention; (2) original studies; (3) human experimentation; (4) studies published in English; (5) chronic interventions with a minimal duration of two weeks; and (6) studies assessing at least VO_2_max (VO_2_max had to be assessed through a sub-maximum or maximum test with gas exchange in an ergometer, and the values from the test had to be expressed as mL/kg/min) or 1-RM (1-RM had to be assessed through the 1-RM test and with a upper- or lower-limb exercise) or body composition of tested participants. Research studies were excluded if they: (1) used a sample population with pathologies, and if they were not between 18 and 65 years of age; (2) were reviews or assessed the effects of an acute intervention; (3) were not an original investigation published in full; (4) did not meet the characteristics of CT (i.e., the sets, volume of rest, etc.); (5) was published in a language other than English; or (6) did not specify the test to be evaluated.

### 2.4. Study Selection and Data Extraction

Retrieved articles were reviewed independently by two authors (JARA and DJRC) in order to select relevant articles. In addition to the literature search, references were scanned for further relevant articles, which were included in our analysis if they met the inclusion criteria. Two authors (JARA and DJRC) independently extracted data from the included studies. The following information was extracted: authors of the paper; study design; country; number of participants included in each group; gender; level of training of the sample; age; height; weight; fat mass (%); body mass index (BMI kg/m^2^); VO_2_max (mL/kg/min); and 1-RM (kg). Regarding the characteristics of the CT intervention, the information extracted included: study duration (weeks); training frequency (days × week); intensity (% RM or other); number of exercises; session duration (min); total number of sessions; number of bouts/sets; number of repetitions; work time in each exercise (seconds); rest between sets (seconds); and rest between exercise (seconds).

### 2.5. Outcomes

The outcomes analyzed were: (a) body composition—(I) body mass, (ii) fat mass (% and kg), and (iii) muscle mass (lean body mass, skeletal muscle mass, and fat-free mass were included as muscle mass components); (b) cardiorespiratory fitness—(i) VO_2_max, (ii) maximum aerobic speed and power (MAS/MAP), (iii) aerobic performance, and (iv) anaerobic threshold; (c) strength, (c1) upper limbs—(i) 1-RM of bench press, (ii) 1-RM of front pull down, (iii) 1-RM of elbow flexion, and (iv) handgrip; (c2) lower limbs—(i) 1-RM of leg press, (ii) 1-RM of leg extension, (iii) 1-RM of half-squat, and (iv) countermovement jump (CMJ) height. If the articles did not include any of these variables, they were removed.

### 2.6. Risk of Bias Assessment (Study Quality)

The methodological quality of the selected studies was assessed with the Cochrane risk-of-bias tool [33] that includes the following domains: (1) random sequence generation (selection bias); (2) allocation concealment (selection bias); (3) blinding of participants and personnel (performance bias); (4) blinding of outcome assessment (detection bias); (5) incomplete outcome data (attrition bias); (6) selective reporting (reporting bias); and (7) other bias. For each study, each item was described as having either a low risk of bias, an unclear risk of bias, or a high risk of bias. Risk of bias was assessed independently by two authors (LAC and DJRC) using the Cochrane risk-of-bias tool [33]. Additionally, a visual inspection of the publication bias was performed using the funnel plot. Egger’s test [34] was also used to analyze the possible publication bias of the funnel plot. The threshold of statistical significance was set at a *p*-value lower than 0.05.

### 2.7. Data Synthesis and Statistical Analysis

The meta-analysis and the statistical analysis were conducted using Review Manager software (RevMan 5.2; Cochrane Collaboration, Oxford, UK). A random effects meta-analysis was conducted to determine the effect of CT on body composition (body mass, fat mass, and muscle mass), cardiorespiratory fitness (VO_2_max, anaerobic threshold, aerobic performance and MAS/MAP), and strength (upper and lower limb 1-RM, handgrip, and CMJ height). Effect sizes of outcomes between training and control arms, as well as the differences before and after training intervention, were calculated as the (adjusted Hedges’ g) standardized mean difference (SMD = $\frac{difference\;in\;mean\;outcome\;between\;groups}{SDpooled=standard\;deviation\;of\;outcome\;among\;participants}$) with mean ± SD and 95% confidence intervals.

$$SMD=\frac{m_{1}-m_{2}}{\sqrt{\frac{(n_{1}-1)\;sd_{1}^{2}+(n_{2}-1)sd_{2}^{2}}{N-2}}}\;\left(1-\frac{3}{4N-9}\right)$$

$$N=(n_{1}+n_{2})$$

The threshold values for SMD were >0.2 (small), >0.5 (moderate), and >0.8 (large) [35]. In addition, the mean difference (MD) was used when all the studies assessed the same outcome and measured it in the same way. Each difference of the means was weighed according to the inverse variance method [36]. 

The heterogeneity between the studies was evaluated through the I^2^ statistic, and between-study variance using the tau-square (Tau^2^) [37]. I^2^ values of 30–60% represented a moderate level of heterogeneity. A *p* < 0.1 value suggests the presence of substantial statistical heterogeneity. The publication bias was evaluated through an asymmetry test as estimated from a funnel plot.

### 2.8. Effects of Moderator Variables: Meta-Regression and Sub-Analysis

Subgroup analysis was performed using Review Manager software (RevMan 5.2; Cochrane Collaboration, Oxford, UK) to analyze the effect of categorical variables. In this case, studies were divided into those analyzing the impact of CT according to: (a) sex (women and men); (b) training status (trained, active, or untrained); (c) intensity (low, <60% 1-RM; moderate, 60–80% 1-RM; and high, >80% 1-RM); (d) training volume (10–20, 21–30, or >30 total sessions); (e) frequency per week (2 or 3 sessions); (f) sets (2, 3, or >3); (g) repetitions (6–12 or >12) and rest between exercise (10–30, 31–60, or >60 s). In each study, the SMD (CI 95%) before and after CT was calculated. The estimate of the effect was estimated using the inverse variance random effects method. The difference between the groups was calculated using the chi-square statistical test (χ^2^). Significance was accepted at an alpha level ≤ 0.05.

## 3. Results

### 3.1. Search Results and Characteristics of Included Studies

**Figure 1:**
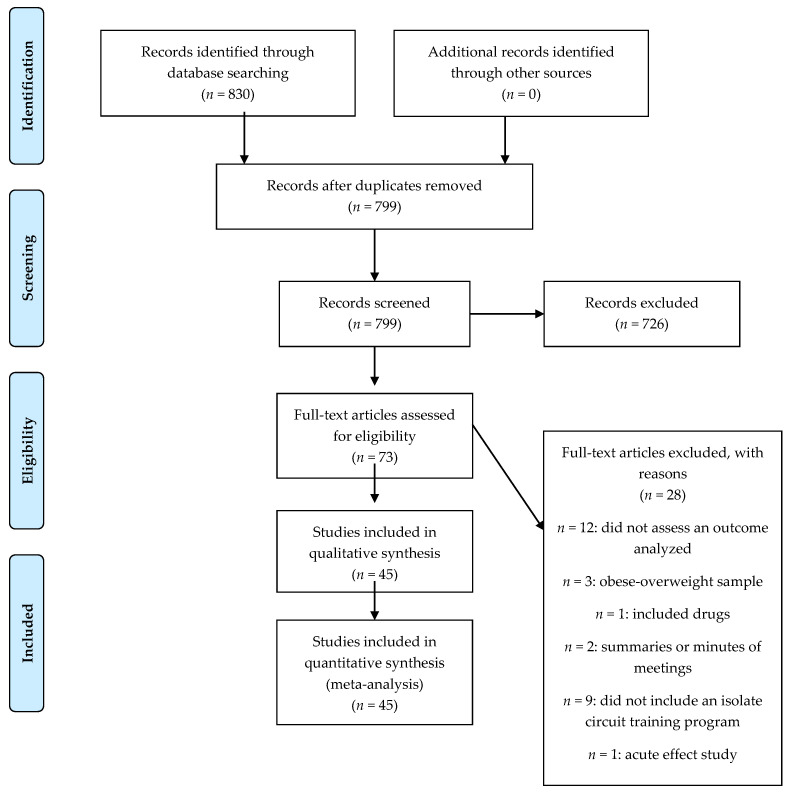
Flow diagram of the included studies.

A total of 830 articles were identified in the initial search. Of these, 799 abstracts and titles were reviewed, 726 articles were deleted, and 73 full-text articles were assessed for eligibility. Finally, 45 studies [10,11,12,19,20,21,22,23,24,25,26,27,28,38,39,40,41,42,43,44,45,46,47,48,49,50,51,52,53,54,55,56,57,58,59,60,61,62,63,64,65,66,67,68,69] met the inclusion and exclusion criteria, including 58 experimental groups (*n* = 897 participants) and 34 control groups (*n* = 474 participants) (Figure 1).

### 3.2. Participants and CT Characteristics

Supplemental Table S1 presents participant details; the range age of the participants was 18–42.5 years, with a weight range of 52.5–95.5 kg and a BMI range of 20.1–30.9 kg/m^2^. Additionally, the CT characteristics are shown in Table 1. The average duration of the CT was 10 weeks (range: 4–28), with a weekly frequency of 2 and 3 sessions per week with between 6 and 14 exercises per session. 

### 3.3. Quality of Studies: Publication Bias

In addition, visual inspection on body composition and endurance outcomes showed an absence of non-significant asymmetry. However, the funnel plots and the Egger test showed a significant asymmetry for the strength outcomes (Supplemental Table S2), except for the CMJ. The publication bias analysis was conducted on the variables included in the subgroup analysis. Supplemental Figure S1 shows the methodological quality of the selected studies assessed with the Cochrane risk-of-bias tool.

### 3.4. Meta-Analysis

#### 3.4.1. Effect of CT on Body Composition

**Figure 2:**
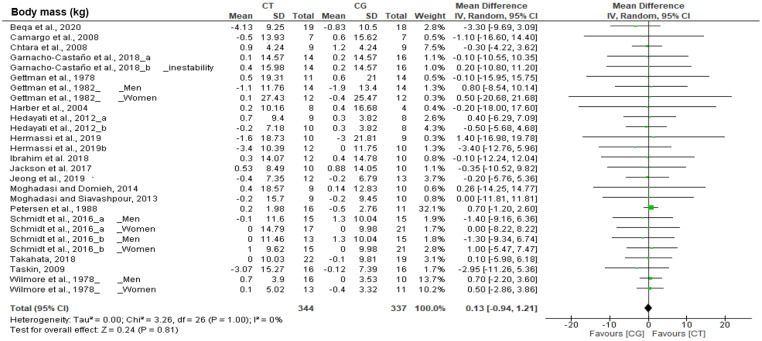

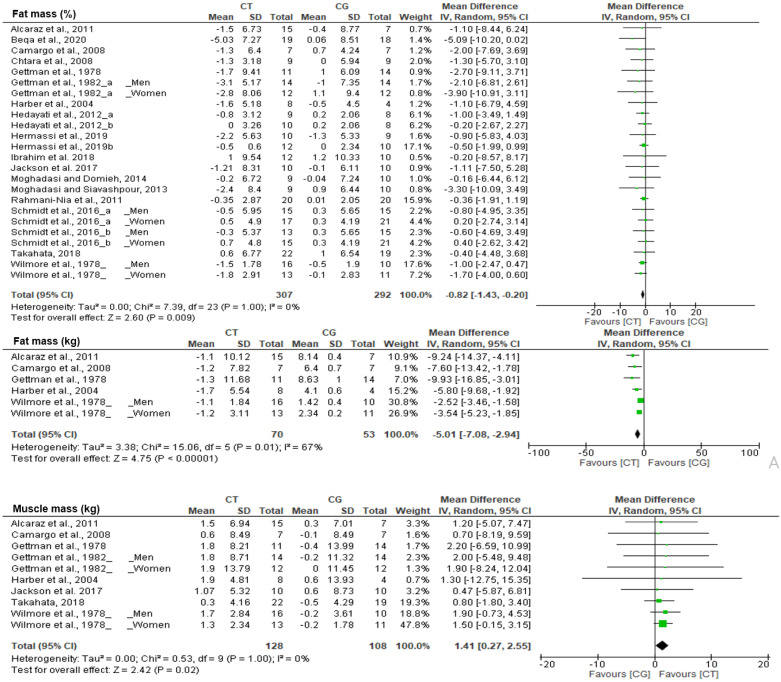
Results of a random effects meta-analysis for resistance circuit training (CT) compared to control group (CG), shown as mean difference and standardized mean difference with 95% CIs on body composition.

Table 2 shows the effects of CT on body composition variables. The CT evoked a significant decrease on fat mass (kg and %: *n* = 395, MD = −0.70%, *p* < 0.001, *I*^2^ = 0%; *n* = 123, MD = −1.04 kg, *p* = 0.004, *I*^2^ = 0%, respectively) and a significant increase on muscle mass after training (*n* =181, MD = 1.18 kg, *p* < 0.001, *I*^2^ = 0%). In addition, significant differences were observed in favor of the CT group when compared to the CG (Figure 2) in fat mass (% and kg) and muscle mass (kg). However, no change in weight was observed. 

#### 3.4.2. Effect of CT on Endurance Outcomes

**Figure 3:**
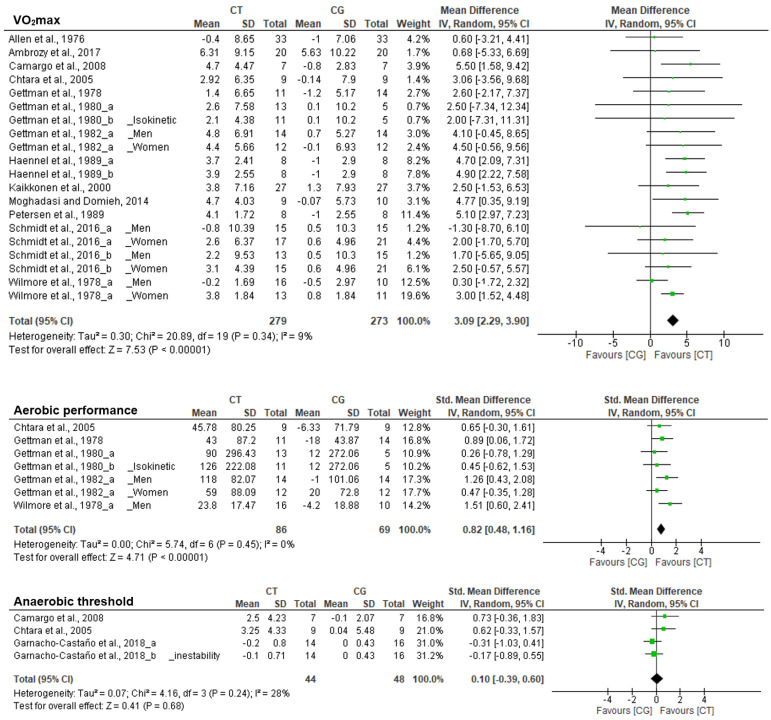
Forest plot showing the results of a random effects meta-analysis for resistance circuit training (CT) compared to control group (CG) on aerobic performance.

The overall effects on VO_2_max (MD: 2.93; IC: 1.97, 3.90), MAS/MAP (MD: 0.35; IC: 0.02, 0.67) and aerobic performance (SMD: 0.65; IC: 0.09, 1.20) showed a significant improvement after CT (Table 3). Additionally, significant differences were found in favor of the CT when compared to the CG on VO_2_max (Z = 7.53; *p* < 0.001) and aerobic performance (Z = 4.71; *p* < 0.001) (Figure 3).

#### 3.4.3. Effect of CT on Strength Outcomes

**Figure 4:**
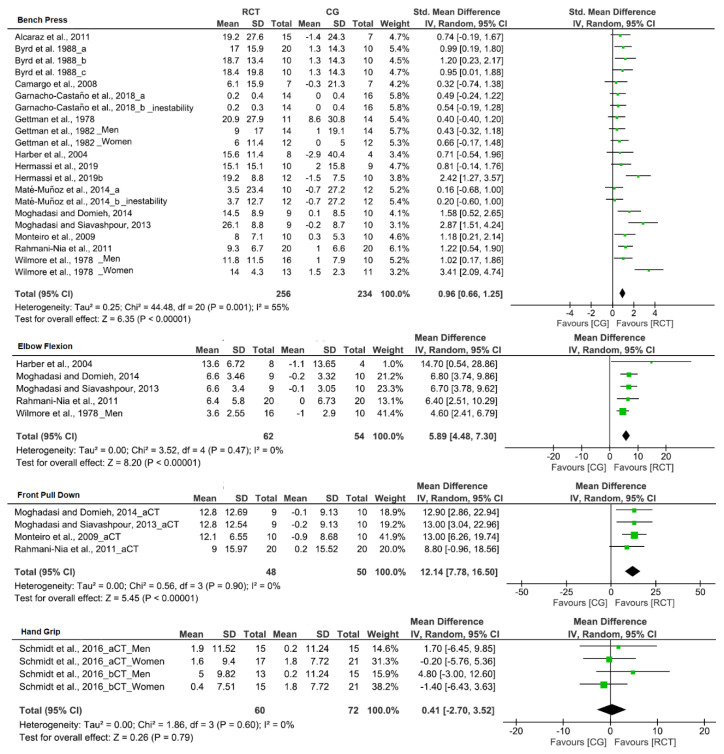
Forest plot showing the results of a random effects meta-analysis for resistance circuit training (CT) compared to control group (CG) on upper limbs exercise.

**Figure 5:**
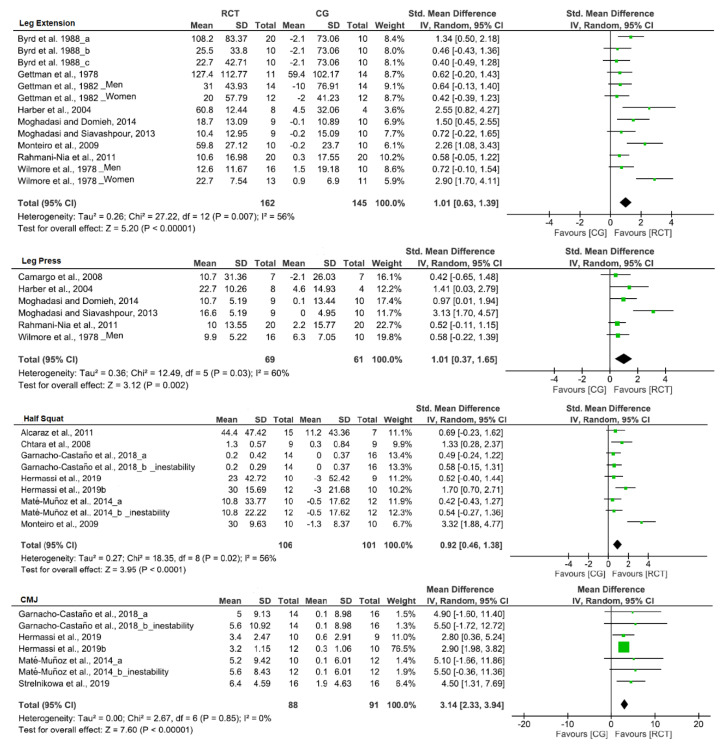
Forest plot showing the results of a random effects meta-analysis for resistance circuit training (CT) compared to control group (CG) on lower limbs exercise.

Table 4 provides the effects of CT on strength outcomes. CT training led to an increase in upper-limb strength (SMD; bench press: 1.16, front pull down: 1.29, elbow flexion: 1.60, handgrip: 0.30) after training. In addition, increases in strength of lower-limb exercise after CT were observed (SMD; leg press: 1.83, leg extension: 1.28, half-squat: 1.20, CMJ: 1.06). Likewise, significant differences were observed in bench press, front pull down, elbow flexion (Figure 4), and lower limbs in leg press, leg extension, half-squat, and CMJ (Figure 5) when compared to CG, in favor of CT group.

### 3.5. Subgroup Analysis

#### 3.5.1. Subgroup Analysis on Body Composition Outcomes

Fat mass: Supplemental Table S3 shows the effect of CT on fat mass according to the level of training of the participants and the training characteristics. Fat mass decrease similarly in trained (MD: −0.52; IC: − 0.86, − 0.18; *p* = 0.003) and untrained participants (MD: −1.22; IC: − 1.80, − 0.65; 4.18). Concerning training characteristics, a greater effect on fat loss was observed when training included 2 or 3 per week, with a total duration between 21–30 (MD: −1.10, CI: −1.65, −0.55) sessions, using low or moderate intensity. Furthermore, sessions which included 3 sets (MD: −0.84; CI: −1.23, −0.45) and >12 repetitions (MD: −1.00; CI: −1.89, −0.10) with differences from 6–12 (χ^2^ = 3.64, *p* = 0.06) and a short rest between exercises (10–30 MD: −1.10, −0.58; IC: −1.63) produced a greater effect in fat decrease. 

Muscle mass: Only untrained participants (SMD: 0.30; IC: 0.04, 0.56) increased muscle mass (Supplementary Table S3) performing CT. A greater effect was observed for the following: when training included 3 sessions per week (SMD: 0.34; CI: 0.11, 0.58), 21–30 sessions (MD: 0.51; CI: 0.17, 0.85), at low intensity (SMD: 0.45; CI: 0.14, 0.77), 3 sets (MD: 0.33; CI: 0.09, 0.58), and short rest between exercise (10–30 s, SMD: 0.44; CI: 0.14, 0.74). 

#### 3.5.2. Subgroup Analysis on Endurance Outcomes

VO_2_max: CT led to a significant increase in VO_2_max in men and women. In addition, VO_2_max increase in trained, active, and untrained participants. Regarding the characteristics of the training, CT produced a significant greater effect on VO_2_max when the program was carried out 3 days a week and had a duration of 21–30 to more than 30 sessions (maximum 112) using moderate or low intensity, and the CT included 2 or 3 sets, from 6–12 to more than 12 repetitions, and used short (10–30 s) or long (>60 s) rest periods. However, no differences between subgroups were found in any of the participants and training characteristics or in the training characteristics (Supplementary Table S5). 

Aerobic performance: No interaction effect was observed on aerobic performance. However, a significant improvement was only observed in studies involving active men that performed 3 sessions per week using more than 30 sessions at low intensity and including 2 sets and more than 12 repetitions and a short rest between exercises (10–30 s). (Supplementary Table S4).

#### 3.5.3. Subgroup Analysis on Strength Outcomes

Bench press: CT led to an increase in bench press strength in trained and untrained participants (χ^2^ = 20.11, *p* < 0.001). In addition, all the characteristics analyzed led to a significant increase after training (Supplementary Table S5).

Leg press: CT improved lower-limb strength (leg press) in men (SMD: 1.15; CI: 0.71, 1.59) and women (MD: 1.65; CI: 0.32, 2.98). In addition, greater significant increases were observed in untrained participants (SMD: 1.74; IC: 1.13, 2.35). Moreover, a greater effect was observed when training included 3 sessions per week (SMD; 1.53; IC: 1.05, 2.02; χ^2^ = 14.12, *p* < 0.001) and a total of 21–30 sessions (SMD; 1.83; IC: 1.16, 2.50; χ^2^ = 15.18, *p* < 0.001). (Supplementary Table S5). 

## 4. Discussion

This systematic review with meta-analysis aimed to assess the effect of CT on body composition, cardiorespiratory fitness, and strength in healthy adults. A secondary objective was also to analyze which training characteristics and dosage had a greater effect in the variables analyzed. The major finding indicates that CT had an overall significant and large effect on body composition, reducing fat mass (average of 4.3%) and increasing muscle mass (average of 1.9%). Additionally, CT significantly improves aerobic performance (i.e., increase maximum aerobic speed and aerobic performance) and VO_2_max (average 6.3%), showing that this type of training increases cardiorespiratory fitness independent of the training protocol used in the studies. In addition, the present meta-analysis showed that CT had an overall significant and large effect on upper (bench press) and lower strength (leg extension) performance (1-RM) (average of 20.0 and 23.0%, respectively). However, only the magnitude of strength performance appears to be influenced by the training (i.e., number of sessions and frequency) and population characteristics (i.e., training status). Moreover, the magnitude of change of fat mass (%) is affected by the training intensity and the rest between exercises. 

The present results clearly show that CT by itself can elicit significant changes in body composition, understood as fat loss and increasing muscle mass, especially in adult men. With regard to fat mass, although most studies have shown significant reductions in body fat with a frequency of training of 2–3 sessions per week, Chtara et al. [25] also showed a decrease of 9.2% in body fat with only 2 sessions per week in active people. In addition, Paoli et al., using similar training volumes and frequency for low-intensity circuit and high-intensity circuit groups, found a greater decrease in body fat in the group that used high intensity [70]. Nevertheless, our results have shown how 3 sessions per week, performed at low or moderate intensities with a higher volume training (more repetitions and shorter rest time) may significantly improve the fat loss in subjects, with no change in the weight. One possible explanation for these findings can be related to the lipolysis linked to low intensity and high volume [71], because of the effect of the fat oxidation optimal range, which has been shown to be reached at around 60% exercise intensity. In addition, these results report an increase in muscle mass (not only muscle mass maintenance) because the body mass was not modified. Hence, the effect of CT on weight could be influenced by the increase of muscle mass and the decrease of fat mass, promoting body mass maintenance. The main reason for this muscle growth may be associated with the intramuscular anabolic signaling, the maximization of the response of muscle fiber recruitment, the time under tension, and the metabolic stress [72] that resistance exercise (e.g., resistance circuit training) promotes. Moreover, differences between untrained and trained subjects have been found in several studies. Since untrained subjects have, among other things, more facility to present changes in body composition, the range of improvement is usually much higher compared to trained subjects, even after just a short period of training [73]. Apart from the training characteristics, this may be due to the fact that, in untrained subjects, exercise induces a larger increase in total energy expenditure than can be attributed to the energy cost of a training program [74]. Therefore, if there is a greater expenditure of energy, the loss of fat mass will be higher.

On the other hand, our meta-analysis extends the evidence that CT interventions are effective for enhancing cardiorespiratory fitness, because there was an overall significant and large pre–post effect of CT training on VO_2_max, MAS/MAP, and aerobic performance, as well as between experimental and control groups. Improvements in VO_2_max are influenced by maximal stroke volume, cardiac output, and peripheral factors (i.e., higher capillarization, improvement in muscle buffering, or increases in activities of metabolic enzymes) [75]. In this way, it has been reported previously that the main responses of the cardiovascular system to resistance circuit training are a significant increase in VO_2_max, with a concomitant improvement in maximal stroke volume and cardiac output [21]. On the other hand, to the best of our knowledge, the effect of CT on the aforementioned peripheral factors have not yet been studied. However, it has been previously reported that the combination of contractile activity, functional hyperemia, low O_2_ tension, and metabolic activity promotes an upregulation of angiogenic factors [76]. Additionally, some previous endurance [77] or resistance training programs [78] found an increase in muscle angiogenesis, showing an increase in vascular endothelial growth factor and capillary-to-fiber ratio. Therefore, although CT is an effective training method to increase cardiorespiratory fitness in healthy adults, little is known about whether it may increase the relative contribution of peripheral factors (i.e., muscle perfusion, mitochondrial capacity, or diffusion) to O_2_ delivery and utilization. Thus, to provide a more complete review and to truly determine the effect of CT, future studies should analyze these aforementioned variables. Remarkably, our results have shown how the training and the population’s characteristics did not influence the magnitude of change in cardiorespiratory fitness variables. However, a statistical trend to obtain a greater effect on these variables was observed in programs with a long duration (>20 sessions), using a frequency of 3 sessions per week and performed at low or moderate intensities with a higher volume training (more repetitions and shorter rest time).

With regard to strength variables, a significant pre–post increase in upper-limb strength was found in bench press, front pull down, elbow flexion, and handgrip exercises, as well as lower-limb exercises (leg press, leg extension, half-squat, and CMJ) after training. In addition, significant differences were observed in both upper- and lower-limb exercises when comparing the CG, in favor of the CT group. One of the main reasons for the increase in strength in the CT group may be due to the significant increase in MM found in the CT group. The increase in strength through structural factors, among which is the increase in muscle mass, is a major pathway for the improvement of muscle strength. The most important adaptations, such as an increase in the cross-sectional area of the whole muscle and individual muscle fibers, is due to an increase in the size and number of myofibrils. Changes in fiber type, muscle architecture, and myofilament density are morphological adaptations, but less important in terms of increasing MM [79].

The studies that led to improvements in upper- and lower-body strength used different management of training variables (frequency of 1 to 3 sessions per week; intensities of 40 to 90% 1-RM; duration of 4 to 28 weeks; total number of sessions of 12 to 112; duration of sessions of 7 to 78 min; number of exercises of 3 to 13; number of sets of 1 to 6; and number of repetitions of 4 to 24). However, in the subgroup analysis, no differences were observed in any of these subgroups in bench press strength, although differences between groups were observed when subdividing by level of training in leg press performance, with greater improvements in the group of untrained participants, as well as when using a frequency of 3 times per week and 21–30 sessions in total. As previous research has concluded, the level of training is a variable that influences adaptations to strength training [80,81]. In this context, those with lower levels of strength may benefit more from CT with the goal of improving strength [50], while those with greater levels of strength may need a more specific stimulus [82]. However, some studies have concluded that CT and traditional strength training may produce similar adaptations in resistance-trained men [11]. Other variables, such as frequency, intensity, and volume, also modify the improvements in muscle mass after strength training. Frequencies of 2 or 3 sessions per week [83] and volumes of at least 10 weekly series per muscle group [84] appear to be adequate to maximize gains in strength and hypertrophy. As for intensity, the literature indicates that, in order to increase strength, high intensities are necessary. However, if the main objective is to increase muscle mass, this can be achieved with both high and low intensities [85].

In this context, untrained individuals will be able to further increase their strength and performance in certain exercises in the early stages of training through neural adaptations (i.e., inter- and intra-muscular coordination, increased recruitment of motor units), regardless of changes in body composition [86]. Even so, that interpretation should be treated with caution since, while differences between groups by level of training have been found in the leg press exercise, no such differences were observed in the bench press. As mentioned above, there is some controversy in the literature about the consequences of the interference effect on strength gains during training such as CT [87]. Therefore, more research is required to compare traditional strength training and CT. Furthermore, research analyzing the molecular basis and the signaling pathways could shed light on this topic, and clarify the true impact of the interference effect.

We acknowledge several limitations of this meta-analysis, which are related in part to the available studies included and the divergent methodologies employed, including (i) the different intensities, volume, and CT characteristic procedures applied in the studies; (ii) the small number of studies using high-intensity resistance circuit training to obtain a more specific picture about the effect of this type of training on body composition, strength, and cardiorespiratory fitness; (iii) the lack of longer studies to analyze the chronic effect of CT (only six studies had a program duration of >12 weeks); and (iv) the lack of nutritional and energy balance control of the included studies, which may affect the individuals’ results of body composition. In addition, we found that the available evidence has a high risk of bias, primarily due to the low quality of available studies. Accordingly, to achieve a more comprehensive picture, future studies should include a better quality of design and analyze the effect of interventions of longer duration. Additionally, future studies should identify the mechanism involved in the improvements in aerobic fitness, strength, and the optimization of body composition after CT. In addition, to provide a more complete review and to truly determine the effect of CT, future systematic reviews with meta-analyses that compare CT with strength training are necessary. From a practical application point of view, if coaches want to obtain greater effects on fat mass loss or VO_2_max improvements, the program should include 3 sessions per week of CT performed at low or moderate intensity and using a high number of repetitions (>20 repetitions) and short periods of rest time (10–30 s), and with a long duration (>20 sessions). However, if the principal aim of the CT program is to obtain greater effects on strength, the program appeared to produce higher gains in untrained participants who performed long duration programs (>20 sessions) with a frequency of 3 sessions per week.

## 5. Conclusions

This systematic review with a meta-analysis concludes that resistance circuit-based training does result in significant reduction in fat mass (average of 4.3%), and it greatly increases muscle mass (average of 1.9%) and upper and lower strength (average 20.0 and 23.0%, respectively). Additionally, CT greatly improves cardiorespiratory fitness (i.e., VO_2_max (average 6.3%), aerobic performance (average 2.6%), and maximum aerobic speed or power (average 0.3%). On the other hand, only the magnitude of strength performance appears to be influenced by the training (i.e., number of sessions and frequency) and population characteristics (i.e., training status). Moreover, the magnitude of change of fat mass (%) is affected by the training intensity and the rest between exercises. CT increases cardiorespiratory fitness independent of the training characteristics and training population used in the studies. 

## Figures and Tables

**Table 1:** Characteristics of the training intervention of studies included in the meta-analysis.

Study	Group	Duration (Weeks)	Frequency (Days x Week)	Intensity (% RM or Other)	Exercises (*n*)	Session Duration (min)	Total Session (*n*)	Bouts/Sets (*n*)	Repetitions (*n*)	Work Time Each Exercise (s)	Rest between Sets (s)	Rest between Exercise (s)
Alcaraz et al., 2011	CT	8	3	85–90	6	55–78	24	3–6	6	25	300	35
	CG	8										
Allen et al., 1976	CT	12	3	75	6	27	36	3	8	30	-	60
	CG	12										
Ambrozy et al., 2017	CT	8	3	-	3–5	60	24	3	15–20	-	120	-
	CG	8	3	-	2	60	24					
Arce-Esquivel and Welsch, 2007	CT1	5	3	60	8	60	15	3	8–12	-	-	-
	CT2	5	3	60	8	60	15	1	8–12	-	-	-
Bachero-Mena et al., 2020	CT	25	1	0–40	7	-	25	3–5	10–30	-	-	-
	RT	25	2	40–55	4	-	50	2–3	4–6	-	-	-
Beqa et al., 2020	CT	8	3	-	8–10	45–60	24	2–3	-	30	300	30
	CG	8										
Byrd et al. 1988	CT1 (no pause)	10	3	75–85	6		30	3	6–10		60	
	CT2 (1 sec rest between reps)	10	3	75–85	6		30	3	6–10		60	
	CT3 (2 sec rest between reps)	10	3	75–85	6		30	3	6–10		60	
	CG	10										
Camargo et al., 2008	CT	12	3	60	-	35	36	3	15	-	-	-
	CG	12										
Chtara et al., 2005	CT	12	2	-	6	30	24	4	-	30–40	120	20–30
	CG	12	-	-	-	-	-	-	-	-	-	-
Chtara et al., 2008	CT	12	2	-	6	30	24	4	-	30–40	120	20–30
	CG	12	-	-	-	-	-	-	-	-	-	-
Dorgo et al., 2009	CT	14	3	67–80	6–9	60	42	2–4	8–12	-	-	20–30
	Manual CT	14	3	67–80	6–9	60	42	2–4	8–12	-	-	20–30
Garnacho-Castaño et al., 2018	CT	8	3	5–8 RPE	8	45–60	24	3	15	-	60–120	0–30
	CT instability	8	3	5–8 RPE	8	45–60	24	3	15	-	60–120	0–30
	CG	8										
Gettman et al., 1978	CT	20	3	50	10	45	60	2	10–20	-	-	20–30
	CG	20										
Gettman et al., 1979	CT	8	3	50–90	8	24	24	2	10–15	-	-	30
Gettman et al., 1980	CT	20	3	50	9	25	60	2	12			30
	CT isokinetic	20	3	60º/s	10	29	60	2	12			30
	CG	20										
Gettman et al., 1982	CT	12	3	40	10	22,5	36	3	12–20	30	-	15
	CG	12										
Getty et al., 2018	CT	4	3	-	6	-	-	3	-	-	-	-
Haennel et al., 1989	CT 1	9	3	60	9	27	27	3	-	20	80	20
	CT 2	9	3	50	9	27	27	3	-	20	80	20
	CG	9										
Harber et al., 2004	CT	10	3	40–60	10	-	30	1–3	12–20	20–30	-	10–30
	CG	10										
Hedayati et al., 2012	CT1	4	4	40	9	50–55	16	3	8–11	30	120	30
	CT2	4	4	80	9	50–55	16	3	8–11	30	120	30
	CG	4										
Hermassi et al., 2019	CT	12	2	80–85 and body weight	6		24	2	6–16		180	180
	CG	12										
Hermassi et al., 2020	CT	10	2	60–80	8		20	2–3	8–12		180	180
	CG	10										
Kaikkonen et al., 2000	CT	12	3	70–80% HR peak	10	40	36	3	-	40	-	20
	CG	12										
Martínez-Guardado et al., 2019	CT	7	2	85	6	60	14	3	6	25	300	35
Maté-Muñoz et al., 2014	CT	7	3	10 RPE	8	45–65	21	3	15	-	60–120	0–30
	CT instability	7	3	10 RPE	8	45–65	21	3	15	-	60–120	0–30
	CG	7										
Messier and Dill, 1985	CT	10	3	67–80/<65	12	20	30	-	8–12	-	-	-
Moghadasi and Domieh, 2014	CT	8	3	65–80	8	50–60	24	2–4	8–12	-	120–180	60–90
	CG	8										
Moghadasi and Siavashpour, 2013	CT	12	3	65–80	8	50–60	36	2–4	8–12	-	120–180	30
	CG	12										
Monteiro et al., 2008	CT	10	3	65–80	7	-	30	3	8–12	-	120	ratio 1:3
	CG	10										
Ibrahim et al., 2018	CT	12	3	-	10	-	30	2–3	-	30	300	60
	CG	12										
Ibrahim et al. 2018b	CT	12	3	Elastic bands	10		36	3	10–20		ratio 1:2	300
	CG	12										
Jackson et al. 2017	CT	4	3	70–80% HRpeak	6	60	12					
	CG	4										
Jeong et al., 2019	CT	10	3	40–60	9	50	30	3	12–20	-	-	20–60
	CG	10										
Petersen et al., 1988	CT	5	4	60% VO_2_max	6		20	2–3	20–24	20	240	20–60
	CG	5	3									
Petersen et al., 1989	CT	6	3,5		10		21	2–3	-	20	240	20–60
	CG	6	3		-		21	-	-	-	-	-
Rahmani-Nia et al., 2011	CT	8	3	45–60	6–8		24	3	12–15	-	180	20
	CG	8										
Ramos-Campo et al., 2018	CT	7	2	85	6	60	14	3	6	25	300	35
Schmidt et al., 2016	CT 1	8	3	-	12	7	24	1	-	30	-	10
	CT 2	8	3	-	12	14	24	2	-	30	-	10
	CG	8										
Sperlich et al., 2018	CT	4	14			6	56					
	CT	4	28			6	112					
Strelnikowa et al., 2019	CT	28	1–2	30–40% body weight or body weight	13			1–5	5–30			
	CG	28										
Taipale et al. 2013	CT	8	1–2		8							ratio 1:3–1:5
Taipale et al. 2014	CT	8	1–2		8		14					ratio 1:3–1:5
	CT	8	1–2		8		14					ratio 1:3–1:5
Takahata, 2018	CT	12	3	-	10	15	36	3	-	30	0	0
	CG	12	-	-	-	-	-	-	-	-	-	-
Taskin, 2009	CT	10	3	75	8		30	3		15	10	40–60
	CG	10	3		8		-	-		-	-	-
Wilmore et al., 1978	CT	10	3	40–55	10	22,5	30	3	-	30		15
	CG	10										

CG = control group, CT = resistance circuit-based training.

**Table 2:** Effect of resistance circuit training and endurance training on body composition outcomes.

	*n* Studies	*n*	Random IV, IC 95%	Test Overall Effects	Heterogeneity			
		Participants	Effects	Z(*p*)	Tau^2^	Chi^2^	*p*	*I* ^2^
Weight (kg)-MD								
CG	24	287	−0.08 [−0.86, 0.70]	0.20 (0.840)	0.000	2.17	1.000	0%
CT	34	456	0.06 [−0.59, 0.72]	0.19 (0.850)	0.000	7.46	1.000	0%
Fat Mass (%)-MD								
CG	22	258	0.02 [−0.45, 0.48]	0.07 (0.950)	0.000	3.43	1.000	0%
CT	29	395	−0.70 [−0.98, −0.43]	5.03 (<0.001)	0.000	23.60	0.700	0%
Fat Mass (kg)-MD								
CG	6	53	− 0.30 [−1.00, 0.41]	0.82 (0.410)	0.000	0.58	0.990	0%
CT	10	123	−1.04 [−1.75, −0.33]	2.87 (0.004)	0.000	0.95	1.000	0%
Muscle Mass (kg)-SMD								
CG	10	108	−0.05 [−0.31, 0.22]	0.34 (0.740)	0.000	0.37	1.000	0%
CT	14	181	0.28 [0.07, 0.49]	2.63 (0.008)	0.000	5.53	0.960	0%

CG = control Group, CT = resistance circuit training, MD = mean differences, SMD = standardized mean difference, IV = inverse variance method, CI = confidence interval.

**Table 3:** Effect of resistance circuit training and endurance training on endurance outcomes.

	*n* Studies	*n*	Random IV, IC 95%	Test Overall Effects	Heterogeneity			
		Participants	Effects	Z(*p*)	Tau^2^	Chi^2^	*p*	*I* ^2^
VO_2_max (mL/kg/min)-MD								
CG	16	234	0.03 [−0.55, 0.61]	0.11 (0.91)	0.000	13.88	0.530	0%
CT	28	367	2.93 [1.97, 3.90]	5.98 (<0.001)	4.010	114.31	<0.001	76%
MAS/MAP-MD								
CG	1	9	0.04 [−0.67, 0.75]	0.11 (0.91)	N/A	N/A	N/A	N/A
CT	5	45	0.35 [0.02, 0.67]	2.07 (0.04)	0.000	3.73	0.440	0%
Aerobic Performance-SMD								
CG	5	64	−0.11 [−0.46, 0.24]	0.63 (0.53)	0.000	3.07	0.690	0%
CT	10	128	0.65 [0.09, 1.20]	2.27 (0.02)	0.620	39.82	<0.001	77%
VT2-SMD								
CG	3	32	−0.01 [−0.50, 0.48]	0.05 (0.96)	0.000	0.01	0.990	0%
CT	5	57	0.20 [−0.29, 0.69]	0.79 (0.43)	0.120	6.63	0.160	40%

CG = control group, CT = resistance circuit training, MD = mean differences, SMD = standardized mean difference, IV = inverse variance method, CI = confidence interval, VT2 = second ventilatory threshold, MAS-MAP = maximum aerobic speed/power.

**Table 4:** Effect of circuit resistance training and endurance training on strength outcomes.

	*n* Studies	*n*	Random IV, IC 95%	Test Overall Effects	Heterogeneity			
		Participants	Effects	Z(*p*)	Tau^2^	Chi^2^	*p*	*I* ^2^
Upper Limbs								
Bench Press-SMD								
CG	17	186	0.11 [−0.09, 0.31]	1.05 (0.290)	0.000	5.15	0.990	0%
CT	27	404	1.16 [0.84, 1.47]	7.21 (0.001)	0.480	102.25	<0.001	75%
Front Pull Down-MD								
CG	4	50	−0.30 [−3.21, 2.60]	0.21 (0.840)	0.000	0.07	0.990	0%
CT	5	61	11.61 [8.83, 14.39]	8.20 (0.001)	0.000	0.78	0.940	0%
Elbow Flexion-MD								
CG	5	54	−0.41 [−1.44, 0.62]	0.79 (0.430)	0.000	0.64	0.960	0%
CT	9	121	5.00 [3.09, 6.92]	5.11 (0.001)	6.970	63.86	<0.001	87%
Hand Grip-MD								
CG	2	36	1.40 [−1.46, 4.25]	0.96 (0.340)	0.000	0.23	0.630	0%
CT	6	95	2.15 [0.01, 4.28]	1.97 (0.050)	0.000	2.48	0.780	0%
Lower Limbs								
Leg Press-SMD								
CG	6	61	0.25 [−0.11, 0.61]	1.34 (0.180)	0.000	4.87	0.430	0%
CT	9	177	1.83 [1.15, 2.52]	5.25 (0.001)	0.790	34.90	<0.001	77%
Leg Extension-SMD								
CG	11	125	0.08 [−0.17, 0.33]	0.65 (0.510)	0.000	4.21	0.940	0%
CT	17	201	1.28 [0.83, 1.74]	5.51 (<0.001)	0.650	62.280	<0.001	74%
Half-Squat-SMD								
CG	7	73	0.02 [−0.31, 0.34]	0.11 (0.910)	0.000	1.80	0.940	0%
CT	11	190	1.20 [0.72, 1.67]	4.95 (0.001)	0.430	38.70	<0.001	74%
CMJ-MD								
CG	5	63	0.43 [−0.16, 1.01]	1.42 (0.150)	0.000	1.85	0.760	0%
CT	11	118	3.58 [2.69, 4.47]	7.87 (0.001)	0.430	12.80	0.240	22%

CG = control group, CT = resistance circuit training, MD = mean differences, SMD = standardized mean difference, IV = inverse variance method, CI = confidence interval.

## Data Availability

The authors confirm that the data supporting the findings of this work are available within the article.

## Supplementary Materials

The following are available online at https://www.mdpi.com/article/10.3390/biology10050377/s1, Figure S1: Risk of Bias of the included studies; Table S1: Characteristics of included studies in the meta-analysis. Table S2: Heterogeneity (Eger’s Test); Table S3: Subgroup analysis of the effect of CT on body composition outcomes; Table S4: Subgroup analysis of the effect of CT on cardiorespiratory fitness outcomes; Table S5: Subgroup analysis of the effect of CT on strength outcomes.
