# Supplementary material for: Effects of Resistance Circuit-Based Training on Body Composition, Strength and Cardiorespiratory Fitness: A Systematic Review and Meta-Analysis
Source: Biology (Basel). 2021 Apr 28;10(5):377. doi: 10.3390/biology10050377 (PMC8145598; doi:10.3390/biology10050377)
Supplement: Supplementary file 1 [file biology-10-00377-s001.zip › biology-1178901-supplementary.pdf]

biology

MDPI

Supplemental

Effects of Resistance Circuit-Based Training on Body Composition, Strength and Cardiorespiratory Fitness: A Systematic Review and Meta-Analysis

Domingo Jesús Ramos-Campo, Luis Andreu Caravaca, Alejandro Martínez-Rodríguez and Jacobo Ángel Rubio-Arias

Supplemental Table 1. Characteristics of included studies in the meta-analysis. .

Study, year	Country	Design	Groups	*n*	Type of Participants	Sex	Age (years)		Weight (kg)		Height (cm)		Fat mass (%)		Body Mass Index (kg/m²)		VO_2max (ml/kg/min or l/min)		1-RM upper limb (kg)		1-RM lower limb (kg)		BC	E	S	CMJ
							Mean	SD	Mean	SD	Mean	SD	Mean	SD	Mean	SD	Mean	SD	Mean	SD	Mean	SD				
Alcaraz et al., 2011	Spain	RCT	CT	15	Trained	M	22,7	± 3,3	75,2	± 8,1	176,0	± 6,0	20,1	± 6.7									x		x	
			CG	7	Trained	M							20,3	± 6,5												
Allen et al., 1976	USA	RCT	CT	33	Untrained	M	18,2		68,0		185,4						46,5	± 6,5						x		
			CG	33	Untrained	M	18,2		72,3		178,8						45,1	± 4,8								
Ambrozy et al., 2017	Poland	RCT	CT	20	Trained	W	27-32										39,5	± 6,4						x		
			CG	20	Trained	W											42,1	± 7,2								
Arce-Esquivel and Welsch, 2007	USA	RCT	CT 1	20	Untrained	M	21,0	± 1,8	74,0	± 14,4	168,0	± 9,6			26,2	± 4,4			56,0	± 36,0	79,4	± 31,6			x	
			CT 2	15	Untrained	M	22,0	± 1,9	85,0	± 28,5	173,0	± 9,4			27,9	± 7,0			60,9	± 32,6	94,0	± 34,4				
Bachero-Mena et al., 2020	Spain	RCT	CT	7	Trained 800-m runners	M	22,6	± 5,9	62,2	± 4,8	172,0	± 5,9														x
Beqa et al., 2020	Kosovo	RCT	CT	19	Untrained	W	23,0	± 3,0	66,5	± 6,5	165,0	± 6,0	34,9	± 5,8	24,02	± 2,4							x			
			CG	18	Untrained	W			64,9	± 7,1			34,0	± 5,9	23,09	± 9,8										

Byrd et al. 1988			CT1 (no pause)	20	Untrained	M	21,1 ± 3,3	72,4 ± 8,8	180,0 ± 4,0		22,3		68,9 ± 10,6	193,6 ± 57,8			x	
			CT2 (1 sec rest between reps)	10	Untrained	M	19,4 ± 3,4	70,4 ± 7,1	176,0 ± 6,0		22,7		62,7 ± 8,5	150,9 ± 23,9				
			CT3 (2 sec rest between reps)	10	Untrained	M	21,1 ± 2,3	76,8 ± 8,3	177,0 ± 4,0		24,5		79,1 ± 15,8	159,1 ± 34,1				
			CG	10	Untrained	M	20,4 ± 2,5	76,5 ± 9,1	177,0 ± 4,0		24,4		68,9 ± 9,2	160,3 ± 49,1				
Camargo et al., 2008	Brasil	RCT	CT	7	Untrained	M	29,0 ± 3,0	84,4 ± 10,0	175,0 ± 7,0	18.4 ± 5,0	27,3 ± 2,0	38,4 ± 2,0	50,3 ± 10,5	68,7 ± 21,2	x	x	x	
			CG	7	Untrained	M	30,0 ± 4,0	95,2 ± 10,0	182,0 ± 6,0	22,0 ± 3,0	28,8 ± 2,0	38,0 ± 2,0	39,4 ± 15,1	60,0 ± 18,7				
Chtara et al., 2005	Tunisia	RCT	CT	9	Active	M	21,4 ± 1,3	68,9 ± 2,9	178,2 ± 5,7	14,2 ± 2,2		50,1 ± 4,9				x		
			CG	9	Active	M		71,5 ± 3,0		14,6 ± 4,0		50,7 ± 6,3						
Chtara et al., 2008	Tunisia	RCT	CT	9	Active	M	21,4 ± 1,3	68,9 ± 2,9	178,2 ± 5,7	14,2 ± 2,2		50,1 ± 4,9			x			
			CG	9	Active	M		71,5 ± 3,0		14,6 ± 4,0		50,7 ± 6,3						
Dorgo et al., 2009	USA	RCT	CT	18	Active	M	27,0 ± 5,7	83,5 ± 17,2	174,4 ± 9,7	21,0 ± 7,3	25,5		67,3 ± 33,2	81,1 ± 35,8			x	
				13	Active	W	23,5 ± 3,9	63,2 ± 8,8	162,9 ± 6,3	29,8 ± 7,5								
			Manual CT	28	Active	M	26,5 ± 6,8	82,7 ± 15,1	174,9 ± 5,7	20,2 ± 7,5	26,1		61,6 ± 34,3	76,7 ± 38,6				
				25	Active	W	24,7 ± 4,9	64,2 ± 10,6	164,8 ± 7,0	29,6 ± 7,2								
Garnacho-Castaño et al., 2018	Spain	RCT	CT	14	Active	M (10) and W (4)	21,3 ± 1,4	66,8 ± 10,3	173,6 ± 9,5		22,1 ± 1,8	3,4 ± 0,8	0,9 ± 0,3	1,1 ± 0,3	x	x	x	x
			CT instability	14	Active	M (12) and W (2)	21,6 ± 3,0	72,9 ± 11,0	175,3 ± 8,6		23,8 ± 2,8	3,6 ± 0,8	0,9 ± 0,2	1,0 ± 0,2				
			CG	16	Active	M (13)	22,0 ± 2,2	73,9 ± 10,3	175,9 ± 8,7		23,9 ± 2,4	3,7 ± 0,7	1,1 ± 0,3	1,0 ± 0,3				

						and W (3)											
Gettman et al., 1978	USA	RCT	CT	11	Active	M	29,0	85,4 ± 13,2	180,0 ±	24,4 ± 6,4		40,0 ± 4,9			x	x	x
			CG	14	Active	M	30,0	84,4 ± 14,9	181,0 ±	23,8 ± 4,4		39,5 ± 3,5					
Gettman et al., 1979	USA	Pre-post	CT	16	Untrained	M	29,00	80,5 ± 6,9	176,0	20,2 ± 3,8	26,0	42,5 ± 4,0	62,6 ± 11,3	144,2 ± 24,0	x	x	x
Gettman et al., 1980	USA	RCT	CT	13	Trained	M	31,3	80,9 ± 7,6		20,8 ± 5,4		55,7 ± 4,2				x	
			CT isokinetic	13	Trained	M		87,0 ± 13,6		23,4 ± 5,6		53,9 ± 3,2					
			CG	7	Trained	M		85,6 ± 12,9		22,1 ± 7,0		54,1 ± 7,6					
Gettman et al., 1982	USA	RCT	CT	14	Active	M	M: 36,1 ± 6,7; W: 35,7 ± 4,9	86,6 ± 8,9	M: 179,9 ± 7,9; W: 166,2 ± 5,3	22,6 ± 3,9	M: 26,8 W: 24,5	40,3 ± 4,2	66,0 ± 11,0	196,0 ± 33,0	x		x
				12	Active	W		67,8 ± 19,2		27,0 ± 5,7		32,8 ± 4,2	30,0 ± 7,0	113,0 ± 29,0			
			CG	14	Active	M		82,7 ± 8,8		22,1 ± 4,5		41,3 ± 2,9	71,0 ± 14,0	216,0 ± 69,0			
				12	Active	W		69,9 ± 18,7		58,5 ± 5,4		45,5 ± 3,9	33,0 ± 4,0	113,0 ± 32,0			
Getty et al., 2018	USA	RCT	CT 1	27	Active	M and W (11%)	36,2 ± 13,7	91,5 ± 17,2		33,5 ± 6,7	30,9 ± 5,9	35,7 ± 4,3			x		
			CT 2	25	Active	M and W (28%)	34,4 ± 15	81,8 ± 20,5		27,9 ± 12	28,8 ± 6,8	37,1 ± 9					
Haennel et al., 1989	Canada	RCT	CT 1	8	Active	M	42,2 ± 2,1	83,2 ± 4,2	178,6 ± 2,4		26,1	32,4 ± 1,6				x	
			CT 2	8	Active	M											
			CG	8	Active	M											
Harber et al., 2004	USA	RCT	CT	8	Untrained	M	23,6 ± 1,8	80,9 ± 8,6		17,6 ± 4,0			104,0 ± 8,7	166,5 ± 9,6	x		x
			CG	4	Untrained	M	20,5 ± 1	81,1 ± 5,4		16,0 ± 3,3			91,5 ± 28,7	118,2 ± 24,9			
Hedayati et al., 2012	Iran	RCT	CT 1	9	Active	W	23,2 ± 1,0	56,6 ± 6,7	163,2 ± 6,2	21,2 ± 2,4	21,2 ± 2,3				x		
			CT 2	10	Active	W	21,9 ± 1,5	55,3 ± 4,5	162,6 ± 4,5	20,6 ± 2,3	20,8 ± 1,6						

			CG	8	Active	W	20,8 ± 1,0	52,6 ± 3,2	161,8 ± 3,4	21,1 ± 1,6	20,1 ± 1,5							
Hermassi et al., 2019	Qatar	RCT	CT	10	Trained	M	18,0 ± 0,9	90,5 ± 13,0	181,0 ± 6,0	17,9 ± 4,3	27,6		83,0 ± 10,6	162,0 ± 33,2	x		x	x
			CG	9	Trained	M	18,0 ± 0,9	85,3 ± 16,3	181,0 ± 6,0	16,2 ± 4,7	26,0		74,7 ± 9,3	156,0 ± 36,0				
Hermassi et al., 2020	Tunisia	RCT	CT	12	Trained	M	20,3 ± 0,5	84,8 ± 7,6	183,0 ± 7,0	13,6 ± 0,6	25,3		73,3 ± 6,2	206,0 ± 12,5	x		x	x
			CG	10	Trained	M	20,1 ± 0,5	80,0 ± 8,5	184,0 ± 7,0	12,7 ± 1,6	23,6		69,5 ± 5,5	205 ± 17,2				
Kaikkonen et al., 2000	Finland	RCT	CT	27	Untrained	M and W (50%)	42,5 ± 7,0	78,7 ± 16,0	170,0 ± 10,0		26,9 ± 3,2	36,6 ± 5,3				x		
			CG	27	Untrained	M and W (50%)	41,9 ± 7,0	73,2 ± 10,0	170,0 ± 10,0		25,4 ± 2,3	37,8 ± 5,3						
Martínez-Guardado et al., 2019	Spain	Pre-post	CT	13	Trained	M	23,2 ± 5,2	69,4 ± 7,4	173,4 ± 6,2	16,3 ± 5,5		55,7 ± 5,0	64,0 ± 14,4	67,1 ± 9,9	x		x	
Maté-Muñoz et al., 2014	Spain	RCT	CT	10	Active	M	21,8 ± 1,1	71,8 ± 6,5	178,4 ± 5,4				78,8 ± 16,2	85,8 ± 26,2			x	x
			CT instability	12	Active	M	21,5 ± 3,0	75,7 ± 9,2	178,0 ± 5,0				77,5 ± 8,2	83,1 ± 13,8				
			CG	12	Active	M	22,3 ± 2,4	75,4 ± 9,9	176,0 ± 7,0				81,7 ± 19,6	78,9 ± 12,8				
Messier and Dill, 1985	USA	RCT	CT	12	Active	M	81,6					45,6 ± 1,7				x		
			TT	11	Active	M	71,3					48,7 ± 1,6						
Moghadasi and Domieh, 2014	Iran	RCT	CT	9	Untrained	M	25,3 ± 2,3	69,2 ± 15,7		16,4 ± 4,7	23,5 ± 3,2	40,5 ± 2,3	68,6 ± 5,6	100,2 ± 9,4	x	x	x	
			CG	10	Untrained	M		67,9 ± 9,0		19,4 ± 5,1	24,99 ± 3,4	36,8 ± 4,0	66,3 ± 5,9	97,2 ± 7,9				
Moghadasi and Siavashpour, 2013	Iran	RCT	CT	9	Untrained	W	25,3 ± 3,2	63,5 ± 11,1		29,7 ± 6,1	24,4 ± 4,9		20,0 ± 5,6	51,4 ± 9,3	x		x	
			CG	10	Untrained	W		58,3 ± 8,2		28,6 ± 4,7	22,5 ± 2,0		21,2 ± 6,3	54,2 ± 11,3				
Monteiro et al., 2009	Brazil	RCT	CT	10	Untrained	W	37,0 ± 1,7	65,2 ± 10,7	157,7 ± 10,8		25,72 ± 3,3						x	

			CG	10	Untrained	W	36,9 ± 1,2	64,5 ± 10,2	158,1 ± 8,9		26,1 ± 2,8						
Ibrahim et al., 2018	Malaysia	RCT	CT	12	Untrained	M	21,0 ± 2,0	60,6 ± 9,9	169,3 ± 7,0	18,3 ± 6,8	21,3 ± 2,8				x		
			CG	10	Untrained	M	22,0 ± 2,0	61,4 ± 10,4	170,4 ± 7,2	18,6 ± 7,3	21,1 ± 2,7						
Ibrahim et al. 2018	Malaysia	RCT	CT	12	Untrained	M	21,0 ± 2,0	60,6 ± 9,9	169,3 ± 7,0	18,3 ± 6,8	21,1 ± 2,7				x		
			CG	10	Untrained	M	22,0 ± 2,0	61,4 ± 10,4	170,4 ± 7,2	18,6 ± 7,3	21,1 ± 2,8						
Jackson et al. 2017	USA	RCT	CT	10	Untrained	W	22,4 ± 4,6	58,1 ± 6,3		32,6 ± 5,9	22,7 ± 2,1	32,5 ± 8,3			x		
			CG	10	Untrained	W	22,5 ± 2,2	57,3 ± 9,8		29,9 ± 4,1	21,2 ± 2,5	±					
Jeong et al., 2019	South Korea	RCT	CT	12	Active	W	24,0 ± 2,0	54,6 ± 5,4	162,0 ± 4,6	20,8 ± 1,9					x		
			CG	13	Active	W	22,0 ± 2,0	53,7 ± 4,7	162,9 ± 3,4	20,2 ± 1,6							
Petersen et al., 1988	Canada	RCT	CT	16	Trained	M	19,4 ± 2,5	73,2 ± 1,4	179,3 ± 1,9		24,3	4,1 ± 0,1			x	x	
			CG	11	Trained	M	21,6 ± 1,2	76,8 ± 2,0	179,4 ± 2,1			4,0 ± 0,1					
Petersen et al., 1989	Canada	RCT	CT	8	Active	M	21,6 ± 0,7	82,30 ± 3,5	184,0 ± 2,3		24,3	52,6 ± 1,0				x	
			CG	8	Active	M	21,1 ± 0,6	82,5 ± 2,8	182,9 ± 1,9			52,4 ± 1,9					
Rahmani-Nia et al., 2011	Iran	RCT	CT	20	Untrained	-	22,6 ± 1,1	72,4 ± 5,6	178,3 ± 0,5	16,4 ± 2,0			66,5 ± 4,5	138,9 ± 12,3	x		x
			CG	20	Untrained	-	22 ± 1,2	74,1 ± 5,25	179,4 ± 0,4	16,2 ± 1,5			63,8 ± 4,8	140,5 ± 12,5			
Ramos-Campo et al., 2018	Spain	Pre-post	CT	13	Trained	M	23,2 ± 5,2	69,4 ± 7,4	173,4 ± 6,2	16,3 ± 5,5		55,7 ± 5,0	64,0 ± 14,4	67,1 ± 9,9		x	
Schmidt et al., 2016	USA	RCT	CT1	15	Active	M	21,5 ± 1,5	79,2 ± 8,2	179,3 ± 4,7	13,6 ± 4,1	24,6 ± 2,0	53,7 ± 7,2			x	x	
				17	Active	W	20,7 ± 1,3	64,4 ± 10,7	166,7 ± 7,6	19,4 ± 3,7	23,2 ± 3,7	41,5 ± 4,4					
			CT2	13	Active	M	20,9 ± 1,0	83,2 ± 7,9	180,8 ± 4,0	12,4 ± 3,9	25,4 ± 2,0	51,4 ± 6,0					
				15	Active	W	20,5 ± 1,5	60,9 ± 6,6	165,3 ± 5,4	18,9 ± 3,3	22,3 ± 1,8	40,3 ± 3,1					
			CG	15	Active	M	21,4 ± 1,1	77,1 ± 7,3	179,1 ± 6,0	11,3 ± 4,1	24,1 ± 2,6	52,7 ± 5,8					
				21	Active	W	20,3 ± 1,1	61,8 ± 7,2	165,2 ± 6,4	19,1 ± 2,8	22,6 ± 2,1	39,2 ± 3,7					
Sperlich et al., 2018	Germany	RCT	CT	12	Untrained	M-W	25,0 ± 5,0	73,1 ± 10,6	176,7 ± 11,2		23,3 ± 1,9	40,9 ± 5,5			x	x	

			CT	12	Untrained	M-W	25,0 ± 5,0	71,2 ± 15,2	172,9 ± 8,4		23,6 ± 3,6	40,1 ± 7,0					
Strelnikowa et al., 2019	Russia	RCT	CT	16	Trained	M											x
			CG	16	Trained	M											
Taipale et al. 2013	Finland	RCT	CT	7	Trained	M	33,7 ± 8,8		180 ± 4,8					180,0	x	x	x
Taipale et al. 2014	Finland	RCT	CT	7	Trained	M	34,0 ± 9,0	84,0 ± 11,0	180,0 ± 5,0		25,9	46,0 ± 6,0		178,0		x	x
			CT	9	Trained	W	35,0 ± 6,0	60,0 ± 7,0	165,0 ± 7,0		22,0	43,0 ± 6,0		122,0			
Takahata, 2018	Japan	RCT	CT	22	Untrained	W	18,5 ± 0,6	50,1 ± 7,1	157,7 ± 5,2	27,3 ± 4,7	20,2 ± 2,4				x		
			CG	19	Untrained	W		52,5 ± 6,9		28,1 ± 4,7	21,0 ± 2,5						
Taskin, 2009	Turkey	RCT	CT	16	Active	M	23,9 ± 1,6	67,0 ± 10,8	172,0 ± 8,0		22,7 ±				x		
			CG	16	Active	M	24,0 ± 1,6	66,5 ± 5,3	174,0 ± 5,0		22,0 ±						
Wilmore et al., 1978	USA	RCT	CT	16	Untrained	M		77,5 ± 2,8	179,3 ± 1,2	13,8 ± 1,3		47,7 ± 1,2	64,2 ± 3,8	184,7 ± 8,2	x	x	x	
				13	Untrained	W		62,7 ± 3,6	164,6 ± 2,0	28,1 ± 2,1		35,5 ± 1,4	29,6 ± 1,2	83,9 ± 4,3				
			CG	10	Untrained	M		71,9 ± 2,6	173,2 ± 2,3	12,1 ± 1,0		48,2 ± 2,2	61,0 ± 5,4	173,6 ± 12,9				
				11	Untrained	W		61,5 ± 2,4	163,9 ± 2,1	29,2 ± 1,9		35,6 ± 1,2	31,2 ± 1,6	86,2 ± 5,4				

CG: Control group; CMJ: countermovement jump; CT: Resistance circuit-based training; E: Endurance variables; M: Men, RCT: Randomized controlled trial; S: Strength variables; W: women.

Supplementary table 2. Heterogeneity (Eger's Test)

Outcomes	***CT groups***			***CT vs Control groups***		
**Body composition**						
	Z	p	Funnel plot	Z	p	Funnel plot
Weight (kg)	-0.726	0.468	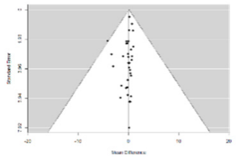	-0.794	0.427	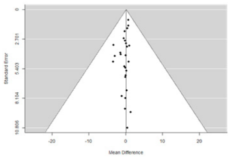
Fat Mass (%)	-1.242	0.214	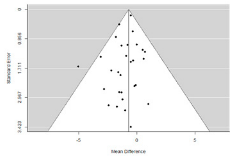	-1.03	0.304	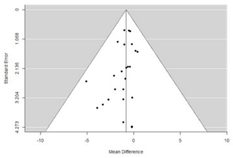
Fat Mass (Kg)	-0.424	0.672	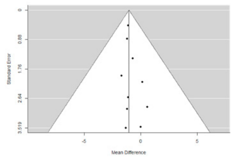	0.228	0.820	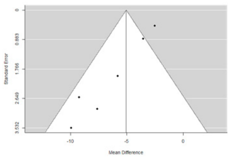
Muscle Mass (kg)	-0.182	0.856	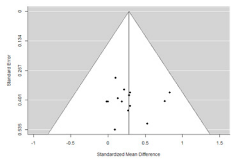	-0.152	0.879	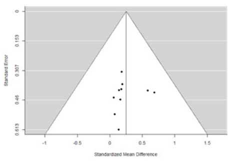
**Endurance Outcomes**						
VO_2max	0.306	0.760	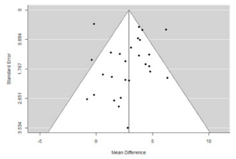	-0.404	0.686	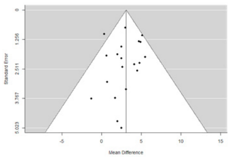
Aerobic Performance	0.776	0.438	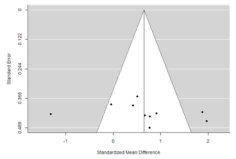	-1.146	0.252	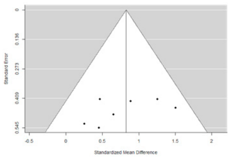
**Strength Outcomes**						

Bench Press	6.291	<0.001	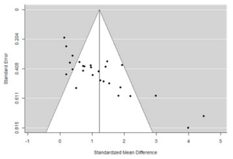	3.921	<0.001	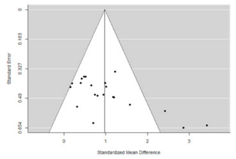
Leg Extension	4.315	<0.001	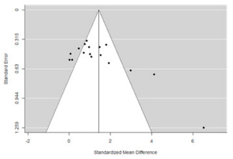	4.112	<0.001	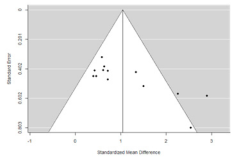
Leg Press	3.633	<0.001	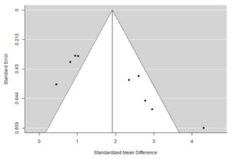	2.368	0.018	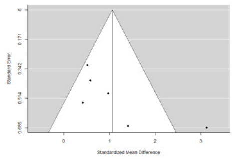
Half-Squat	4.335	<0.001	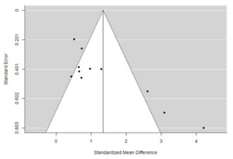	3.932	<0.001	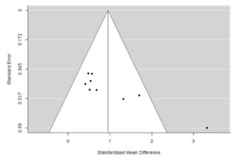
CMJ	0.796	0.426	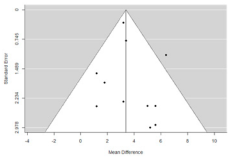	1.457	0.145	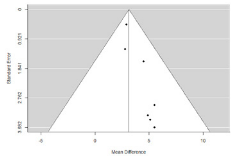

CT: Resistance circuit-based training; CMJ: Countermovement jump.

0

Supplemental table 3. Subgroup analysis of the effect of CT on body composition outcomes

	n studies	n participants	Random IV, IC 95% Effects	Test overall effects Z(p)	Heterogeneity Tau²	Chi²	p	I^2	Difference between groups. Chi² (p)
**Fat Mass (%) - MD**									
Sex									
Men	19	228	-0.68 [-0.97, -0.39]	4.55 (<0.001)	0.000	10.8	0.900	0%	0.07 (0.79)
Women	9	127	-0.83 [-1.92, 0.25]	1.51 (0.130)	0.980	12.9	0.110	38%	
Training status									
Trained	5	57	-0.52 [-0.86, -0.18]	3.00 (0.003)	0.000	1.1	0.890	0%	4.54 (0.10)
Active	12	177	-0.68 [-1.47, 0.11]	1.69 (0.090)	0.000	7.3	0.780	0%	
Untrained	12	161	-1.25 [-1.83, -0.67]	4.24 (<0.001)	0.000	10.7	0.470	0%	
Frequency (days x week)									
2	5	51	-0.54 [-0.87, -0.20]	3.12 (0.002)	0.000	1.5	0.830	0%	3.59 (0.06)
3	22	325	-1.12 [-1.63, -0.62]	4.436 (<0.001)	0.000	18.0	0.650	0%	
Total Session (number)									
10 - 20	5	54	-0.49 [-0.83, -0.16]	2.91 (0.004)	0.000	0.4	0.950	0%	4.38 (0.11)
21 - 30	14	195	-1.10 [-1.65, -0.55]	3.92 (<0.001)	0.050	13.5	0.410	4%	
>30	8	87	-1.41 [-2.89, 0.08]	1.86 (0.06)	0.000	4.7	0.580	0%	
Intensity (% RM)									
Low	9	115	-1.31 [-1.89, -0.72]	4.39 (<0.001)	0.000	5.8	0.660	0%	5.89 (0.05)
Moderate	6	66	-0.50 [-0.84, -0.17]	2.94 (0.003)	0.000	0.9	0.970	0%	
High	3	38	-1.40 [-3.75, 0.95]	1.16 (0.240)	0.000	0.6	0.750	0%	
Sets									
<2	8	105	-0.40 [-1.45, 0.65]	0.75 (0.450)	0.000	3.2	0.870	0%	2.36 (0.31)
3	17	249	-0.96 [-1.46, -0.46]	3.73 (<0.001)	0.160	19.6	0.240	19%	
>3	2	24	-1.33 [-3.24, 0.57]	1.37 (0.170)	0.000	0.0	0.940	0%	
Repetitions									
6 - 12	8	77	-0.49 [-0.81, -0.17]	3.01 (0.003)	0.000	1.1	0.990	0%	3.64 (0.06)
>12	8	90	-1.79 [-3.09, -0.50]	2.71 (0.007)	0.000	2.6	0.920	0%	
Rest between exercises									
10 -30	15	197	-1.10 [-1.63, -0.58]	4.11 (0.001)	0.080	17.1	0.380	6%	5.92 (0.05)
31 - 60	2	28	-0.73 [-3.92, 2.46]	0.45 (0.66)	0.000	0.2	0.660	0%	
>60	5	55	-0.51 [-0.85, -0.17]	2.92 (<0.001)	0.000	1.2	0.750	0%	
**Muscle Mass - SMD**									
Sex									
Men	8	100	0.26 [-0.05, 0.56]	1.67 (0.100)	0.000	2.52	0.93	0%	0.05 (0.83)
Women	4	57	0.29 [-0.08, 0.66]	1.54 (0.120)	0.000	1.8	0.62	0%	
Training status									

Trained	2	28	0.22 [-0.30, 0.75]	0.83 (0.410)	0.000	0.09	0.76	0%	
Active	3	37	0.26 [-0.20, 0.72]	1.10 (0.270)	0.000	0.04	0.98	0%	0.08 (0.96)
Untrained	9	116	0.30 [0.04, 0.56]	2.26 (0.020)	0.000	5.31	0.72	0%	
Frequency (days x week)									
2	3	37	0.04 [-0.41, 0.50]	0.18 (0.86)	0.000	0.09	0.96	0%	1.33 (0.25)
3	11	144	0.34 [0.11, 0.58]	2.87 (0.004)	0.000	4.11	0.94	0%	
Total Session (number)									
10 - 20	2	23	0.19 [-0.39, 0.77]	0.66 (0.510)	0.000	0.05	0.82	0%	
21 - 30	5	68	0.51 [0.17, 0.85]	2.91 (0.004)	0.000	2.13	0.71	0%	2.76 (0.25)
>30	7	90	0.13 [-0.16, 0.43]	0.90 (0.370)	0.000	0.59	1	0%	
Intensity (% RM)									
Low	6	74	0.49 [0.16, 0.82]	2.91 (0.004)	0.000	2.32	0.58	0%	
Moderate	3	33	0.21 [-0.28, 0.69]	0.84 (0.400)	0.000	0.007	0.97	0%	1.24 (0.54)
High	2	28	0.22 [-0.30, 0.75]	0.83 (0.41)	0.000	0.09	0.76	0%	
Sets									
<2	3	35	0.31 [-0.16, 0.79]	1.30 (0.190)	0.000	0.25	0.88	0%	
3	6	75	0.41 [0.09, 0.74]	2.48 (0.01)	0.000	3.27	0.66	0%	0.16 (0.92)
>3	1	15	0.30 [-0.42, 1.02]	0.81 (0.420)					
Repetitions									
6 - 12	2	28	0.22 [-0.30, 0.75]	0.83 (0.410)	0.000	0.09	0.76	0%	0.02 (0.90)
>12	6	68	0.26 [-0.08, 0.60]	1.52 (0.130)	0.000	0.43	0.99	0%	
Rest between exercises									
10 -30	7	90	0.44 [0.14, 0.74]	2.90 (0.004)	0.000	2.8	0.83	0%	0.50 (0.48)
30 - 60	2	28	0.22 [-0.30, 0.75]	0.83 (0.410)	0.000	0.9	0.76	0%	

Supplemental table 4. Subgroup analysis of the effect of CT on cardiorespiratory fitness outcomes

	n studies	n participants	Random IV, IC 95% Effects	Test overall effects Z(p)	Heterogeneity Tau²	Chi²	p	I^2	Difference between groups. Chi² (p)
**VO_2max MD**									
Sex									
Men	19	230	2.74 [1.43, 4.05]	4.09 (<0.001)	5.710	104.13	<0.001	83%	1.57 (0.21)
Women	6	86	3.72 [2.91, 4.54]	8.93 (<0.001)	0.000	2.98	0.700	0%	
Training status									
Trained	7	80	2.62 [1.06, 4.18]	3.30 (<0.001)	0.000	4.54	0.600	0%	
Active	12	142	3.86 [2.93, 4.80]	8.08 (<0.001)	1.040	20.32	0.040	46%	3.61 (0.16)
Untrained	9	145	2.21 [0.45, 3.96]	2.46 (0.010)	5.060	50.00	<0.001	84%	
Frequency (days x week)									
2	7	69	1.59 [-0.31, 3.50]	1.64 (0.100)	0.000	1.40	0.970	0%	4.93 (0.09)
3	20	290	3.09 [1.93, 4.25]	5.21 (<0.001)	4.920	107.04	<0.001	82%	
Total Session (number)									
10 - 20	4	36	1.55 [-1.10, 4.21]	1.15 (0.25)	0.000	0.51	0.920	0%	
21 - 30	14	179	3.26 [1.89, 4.63]	4.67 (0.001)	5.080	101.78	<0.001	87%	1.34 (0.51)
>30	10	152	2.65 [1.46, 3.85]	4.37 (<0.001)	0.590	10.74	0.290	16%	
Intensity (% RM)									
Low	9	106	2.88 [1.28, 4.49]	3.52 (<0.001)	4.390	52.24	<0.001	85%	
Moderate	5	104	3.50 [1.32, 5.67]	3.15 (0.002)	5.690	25.36	<0.001	88%	1.68 (0.43)
High	1	13	0.60 [-3.21, 4.41]	0.31 (0.760)					
Sets									
<2	8	111	2.12 [1.01, 3.22]	3.75 (<0.001)	0.000	2.62	0.920	0%	
3	13	188	3.31 [1.98, 4.63]	4.88 (<0.001)	4.310	74.56	<0.001	84%	1.84 (0.40)
>3	1	9	2.92 [-1.23, 7.07]	1.38 (0.170)					
Repetitions									
6 - 12	6	91	2.84 [0.39, 5.30]	2.27 (0.020)	7.190	27.05	<0.001	82%	0.26 (0.61)
>12	6	80	3.61 [1.97, 5.26]	4.30 (<0.001)	1.300	7.24	0.200	31%	
Rest between exercises (seconds)									
10 -30	16	218	2.68 [1.57, 3.80]	4.72 (<0.001)	3.070	56.31	<0.001	73%	
31 - 60	3	54	1.71 [-1.57, 4.98]	1.02 (0.310)	6.470	9.70	0.008	79%	2.45 (0.29)
>60	1	9	4.70 [2.07, 7.33]	4.72 (<0.001)					
**Aerobic Performance SMD**									
Sex									
Men	8	103	0.84 [0.36, 1.32]	3.44 (0.006)	0.300	18.58	0.010	62%	0.83 (0.36)
Women	2	25	-0.20 [-2.38, 1.98]	0.18 (0.860)	2.290	13.04	<0.001	92%	
Training status									

Trained	3	37	0.35 [-0.11, 0.81]	1.48 (0.140)	0.000	1.91	0.390	0%	
Active	4	46	1.07 [0.49, 1.65]	3.63 (<0.001)	0.140	4.97	0.170	40%	3.73 (0.16)
Untrained	3	45	0.36 [-1.34, 2.06]	0.42 (0.680)	2.090	26.93	<0.001	93%	
Frequency (days x week)									
2	2	22	0.31 [-0.47, 1.09]	0.77 (0.440)	0.130	1.64	0.200	39%	0.61 (0.44)
3	8	106	0.72 [0.05, 1.40]	2.09 (0.040)	0.760	36.69	<0.001	81%	
Total Session (number)									
10 - 20	1	13	-0.04 [-0.81, 0.73]	0.10 (0.920)					
21 - 30	4	54	0.46 [-0.81, 1.73]	0.71 (0.480)	1.480	27.40	<0.001	89%	4.23 (0.12)
>30	5	61	0.92 [0.41, 1.43]	3.56 (<0.001)	0.140	6.99	0.140	43%	
Intensity (% RM)									
Low	7	90	0.72 [0.05, 1.40]	2.09 (0.040)	0.760	36.69	<0.001	91%	
Moderate	1	16	0.51 [-0.19, 1.22]	1.42 (0.150)					2.11 (0.35)
High	1	13	-0.04 [-0.81, 0.73]	0.10 (0.920)					
Sets									
<2	4	51	0.57 [0.18, 0.97]	2.83 (0.005)	0.000	0.43	0.963	0%	
3	5	68	0.68 [-0.51, 1.87]	1.12 (0.260)	1.650	39.26	<0.001	90%	0.14 (0.93)
>3	1	9	0.77 [-0.20, 1.73]	1.56 (0.12)					
Repetitions									
6 - 12	3	37	0.35 [-0.11, 0.81]	1.48 (0.140)	0.000	1.91	0.390	0%	2.56 (0.11)
>12	4	53	0.98 [0.36, 1.59]	3.12 (0.002)	0.210	6.56	0.090	54%	
Rest between exercises									
10 -30	9	115	0.73 [0.12, 1.33]	2.35 (0.020)	0.670	36.71	<0.001	78%	2.34 (0.13)
31 - 60	1	13	-0.04 [-0.81, 0.73]	0.10 (0.920)					

Supplemental Table 5. Subgroup analysis of the effect of CT on strength outcomes

	n studies	n participants	Random IV, IC 95%	Test overall effects	Heterogeneity				Difference between groups. Chi² (p)
			Effects	Z(p)	Tau²	Chi²	p	I^2	
**Bench Press (SMD)**									
Sex									
Men	20	279	1.06 [0.75, 1.37]	6.72 (<0.001)	0.300	51.88	<0.001	63%	1.60 (0.210)
Women	5	97	2.00 [0.57, 3.43]	2.75 (0.006)	2.300	48.19	<0.001	92%	
Training status									
Trained	4	50	1.53 [0.76, 2.29]	3.91 (<0.001)	0.370	7.99	0.050	62%	20.11 (<0.001)
Active	9	171	0.44 [0.21, 0.66]	3.74 (0.0002)	0.010	8.57	0.380	7%	
Untrained	14	183	1.53 [1.03, 2.04]	5.96 (<0.001)	0.670	53.82	<0.001	76%	
Frequency (days x week)									
2	3	35	1.77 [0.76, 2.79]	3.43 (<0.001)	0.530	6.00	0.050	67%	1.64 (0.200)
3	24	369	1.08 [0.76, 1.40]	6.54 (<0.001)	0.450	88.51	<0.001	74%	
Total Session (number)									
10 - 20	4	60	1.11 [0.15, 2.07]	2.27 (0.020)	0.770	16.61	<0.001	82%	3.15 (0.210)
21 - 30	16	207	1.33 [0.96, 1.70]	7.03 (<0.001)	0.350	40.97	<0.001	63%	
>30	7	137	0.74 [0.20, 1.28]	2.71 (0.007)	0.340	21.90	0.001	73%	
Intensity (% RM)									
Low	9	122	1.39 [0.85, 1.93]	5.04 (<0.001)	0.460	26.92	<0.001	70%	2.94 (0.230)
Moderate	13	222	1.15 [0.66, 1.64]	4.62 (<0.001)	0.590	60.44	<0.001	80%	
High	5	60	0.81 [0.38, 1.23]	3.72 (0.0002)	0.050	4.98	0.290	20%	
Sets									
<2	5	60	0.89 [0.40, 1.38]	3.58 (<0.001)	0.110	6.19	0.190	35%	1.30 (0.520)
3	21	329	1.24 [0.85, 1.63]	6.22 (<0.001)	0.610	95.97	<0.001	79%	
>3	1	15	0.96 [0.20, 1.72]	2.47 (0.010)	N/A	N/A	N/A	N/A	
Repetitions									
6 - 12	12	215	1.09 [0.63, 1.55]	4.62 (< 0.00001)	0.470	48.59	<0.001	77%	0.07 (0.790)
>12	13	160	1.01 [0.65, 1.37]	5.46 (< 0.00001)	0.240	26.68	0.009	55%	
Rest between exercises									
10 -30	15	253	1.09 [0.64, 1.53]	4.79 (<0.001)	0.570	68.82	<0.001	80%	1.67 (0.430)
31 - 60	3	37	1.31 [0.68, 1.94]	4.09 (<0.001)	0.090	2.85	0.240	30%	
61 -90	2	22	2.13 [0.54, 3.73]	3.95 (<0.001)	1.010	4.12	0.040	76%	
31 - 60	N/A	N/A	N/A	N/A	N/A	N/A	N/A	N/A	
61 -90	2	22	3.23 [2.64, 3.82]	10.65 (<0.001)	0.000	0.06	0.810	0%	
**Leg Extension (SMD)**									
Sex									

Men	12	148	1.15 [0.71, 1.59]	5.07 (<0.001)	0.370	30.79	0.001	64%	0.49
Women	5	53	1.65 [0.32, 2.98]	2.43 (0.020)	1.980	31.42	<0.001	87%	(0.480)
Training status									
Trained	3	23	0.08[-0.50, 0.66]	0.28 (0.780)	0.000	0.03	0.980	0%	15.03
Active	3	37	0.95 [0.37, 1.52]	3.23 (0.001)	0.070	0.03	0.980	26%	(<0.001)
Untrained	11	141	1.74 [1.13, 2.35]	5.61 (<0.001)	0.750	42.65	<0.001	77%	
Frequency (days x week)									
2	3	23	0.08[-0.50, 0.66]	0.28 (0.780)	0.000	0.03	0.980	0%	14.12
3	14	178	1.53 [1.05, 2.02]	6.18 (<0.001)	0.590	48.06	<0.001	73%	(<0.001)
Total Session (number)									
10 - 20	3	23	0.08[-0.50, 0.66]	0.28 (0.780)	0.000	0.03	0.980	0%	15.18
21 - 30	10	132	1.83 [1.16, 2.50]	5.37 (<0.001)	0.840	42.22	<0.001	79%	(<0.001)
>30	4	46	0.96 [0.52, 1.40]	4.28 (<0.001)	0.650	62.28	<0.001	74%	
Intensity (% RM)									
Low	8	104	1.95 [1.10, 2.80]	4.50 (<0.001)	1.150	40.89	<0.001	83%	2.47
Moderate	6	74	1.19 [0.77, 1.61]	5.59 (<0.001)	0.060	6.58	0.250	24%	(0.120)
High	N/A	N/A	N/A	N/A	N/A	N/A	N/A	N/A	
Sets									
<2	3	35	2.36 [0.42, 4.29]	2.38 (0.020)	2.330	15.87	<0.001	87%	1.28
3	9	114	1.21 [0.79, 1.63]	5.59 (<0.001)	0.210	16.20	0.040	51%	(0.260)
>3	N/A	N/A	N/A	N/A	N/A	N/A	N/A	N/A	
Repetitions									
6 - 12	6	68	1.50 [0.93, 2.08]	5.09 (<0.001)	0.260	10.18	0.070	51%	0.44
>12	6	81	1.19 [0.49, 1.90]	3.33 (<0.001)	0.520	18.46	0.002	73%	(0.500)
Rest between exercises									
10 -30	9	119	1.52 [0.86, 2.19]	4.50 (<0.001)	0.750	36.70	<0.001	78%	
31 - 60	1	9	1.93 [0.76, 3.09]	3.24 (0.001)	N/A	N/A	N/A	N/A	0.35 (0.56)
61 -90	N/A	N/A	N/A	N/A	N/A	N/A	N/A	N/A	

	Random sequence generation (selection bias)	Allocation concealment (selection bias)	Blinding of participants and personnel (performance bias)	Blinding of outcome assessment (detection bias)	Incomplete outcome data (attrition bias)	Selective reporting (reporting bias)	Other bias
Alcaraz et al., 2011	+	?	−	?	+	+	+
Allen et al., 1976	?	?	−	?	+	+	+
Ambrozy et al., 2017	+	?	−	?	+	+	+
Arce-Esquivel and Welsch, 2007	+	?	−	?	+	+	+
Bachero-Mena et al., 2020	−	−	−	?	?	+	+
Beqa et al., 2020	−	−	−	?	+	+	+
Byrd et al., 1988	−	−	−	?	+	+	+
Camargo et al., 2008	+	?	−	?	+	+	+
Chtara et al., 2005	?	−	−	?	+	+	+
Chtara et al., 2008	?	−	−	?	+	+	+
Dorgo et al., 2009	+	?	−	?	+	+	+
Garnacho-Castaño et al., 2018	+	?	−	?	+	+	+
Gettman et al., 1978	+	?	−	?	+	+	+
Gettman et al., 1979	−	−	−	?	+	+	+
Gettman et al., 1980	?	?	−	?	+	+	+
Gettman et al., 1982	+	?	−	?	+	+	+
Getty et al., 2018	?	?	−	?	+	+	+
Haennel et al., 1989	?	?	−	?	+	+	+
Harber et al., 2004	+	?	−	?	+	+	+
Hedayati et al., 2019	+	?	−	?	+	+	+
Hermassi et al., 2019	+	?	−	−	+	+	+
Hermassi et al., 2019b	+	?	−	+	+	+	+
Ibrahim et al., 2018	+	?	−	+	+	+	+
Ibrahim et al., 2018b	+	?	−	?	+	+	+
Jackson et al., 2017	+	?	−	+	−	?	+
Jeong et al., 2019	+	?	−	?	+	+	+
Kaikkonen et al., 2000	+	?	−	+	+	+	+
Martínez-Guardado et al., 2019	+	?	+	+	+	+	+
Maté-Muñoz et al., 2014	+	?	−	?	+	+	+
Messier and Dill, 1985	−	−	−	?	+	+	+
Moghadasi and Domieh, 2014	+	?	−	?	+	+	+
Moghadasi and Siavashpour, 2013	+	?	−	?	+	+	+
Monteiro et al., 2019	+	?	−	?	+	+	+
Petersen et al., 1988	?	?	−	?	+	+	+
Petersen et al., 1989	?	?	−	?	+	+	+
Rahmani-Nia et al., 2011	+	?	−	?	+	+	+
Ramos-Campo et al., 2018	+	?	+	?	+	+	+
Schmidt et al., 2016	+	?	−	?	+	+	+
Sperlich et al., 2018	+	?	−	?	+	+	+
Strelnikowa et al., 2019	?	?	−	?	+	+	+
Taipale et al., 2013	?	?	−	?	+	+	+
Taipale et al., 2014	?	?	−	?	+	+	+
Takahata, 2018	?	?	−	?	+	+	+
Taskin, 2009	+	?	−	?	+	+	+
Wilmore et al., 1978	−	−	−	?	+	+	+

Supplemental Figure 1: Risk of Bias of the included studies
